# SMFC as a tool for the removal of hydrocarbons and metals in the marine environment: a concise research update

**DOI:** 10.1007/s11356-021-13593-3

**Published:** 2021-04-23

**Authors:** Edvige Gambino, Kuppam Chandrasekhar, Rosa Anna Nastro

**Affiliations:** 1 Department of Biological Sciences, University of Naples “Federico II”, Complesso Universitario di Monte Sant’Angelo, Naples, Italy; 2 Department of Biotechnology, National Institute of Technology Warangal, Telangana, 506004 India; 3 Department of Science and Technology, University Parthenope of Naples, Naples, Italy

**Keywords:** Microbial fuel cells, Marine pollution, Hydrocarbons, Heavy metals, Sediment remediation, Renewable energy systems

## Abstract

Marine pollution is becoming more and more serious, especially in coastal areas. Because of the sequestration and consequent accumulation of pollutants in sediments (mainly organic compounds and heavy metals), marine environment restoration cannot exempt from effective remediation of sediments themselves. It has been well proven that, after entering into the seawater, these pollutants are biotransformed into their metabolites, which may be more toxic than their parent molecules. Based on their bioavailability and toxic nature, these compounds may accumulate into the living cells of marine organisms. Pollutants bioaccumulation and biomagnification along the marine food chain lead to seafood contamination and human health hazards. Nowadays, different technologies are available for sediment remediation, such as physicochemical, biological, and bioelectrochemical processes. This paper gives an overview of the most recent techniques for marine sediment remediation while presenting sediment-based microbial fuel cells (SMFCs). We discuss the issues, the progress, and future perspectives of SMFC application to the removal of hydrocarbons and metals in the marine environment with concurrent energy production. We give an insight into the possible mechanisms leading to sediment remediation, SMFC energy balance, and future exploitation.

## Introduction

### Marine pollution: a hot issue

With the advance of modern civilization, the marine environment had been subjected to significant impacts due to agriculture, sewage and industrial effluents drain, urban runoff, atmospheric depositions of different pollutants, oil spillages, mariculture and shipping practices, and operational discharges (Tornero and Hanke 2016). Before 1972, in the USA, waste was frequently dumped in coastal and ocean waters, based on the assumption that seawater had an unlimited capacity to mix and disperse both materials and effluents (EPA 2020). With the London Protocol, in 2006, 51 countries agreed to forbid waste dumping in sea areas. Nevertheless, both solid waste and toxic chemicals are increasingly contaminating the marine environment. If the effects of compounds like polychlorinated biphenyls (PCB) and polycyclic aromatic hydrocarbons (PAHs) had been thoroughly investigated and, at least, in part ascertained (Bhaskar Reddy et al. 2019; Santana et al. 2018), less is known about the consequence of solid waste disposal on the marine environment. Solid waste such as plastics, pieces of furniture, and tires proved to affect sea resources and ecosystems by augmenting the natural flow of nutrients, metals, and other materials to the ocean (Burroughs 1999). Furthermore, there are evidences of a release of toxic compounds like phthalates (able to harm both human health and environment (Paluselli et al. 2019; Mankidya et al. 2013), small polymers, and other organic molecules whose effects on marine life forms are far to be assessed (Gewert et al. 2015). While PAHs are produced by both natural and anthropogenic sources (Howsam and Jones 1998; Manahan 2000), heavy metals spread in the environment due to leakages from chemical and/or petrochemical industries, harbour or military areas, urban, agricultural, or mining settlements, etc.(Furness 2018; Trifuoggi et al. 2017). In 1991, the US Environmental Protection Agency (EPA) classified 16 among PAHs and 12 among heavy metals (As, Be, Cd, Cr, Cu, Pb, Hg, Ni, Se, Ag, Ti, and Zn) as priority pollutants (Santana et al. 2018). Marine sediments play a role in accumulating and transporting contaminants acting as a secondary source of pollution, especially in the coastal area (Everaert et al. 2017). Sediment texture, reduction/oxidation state, adsorption/desorption kinetics, physical transport, and absorption from the water column affect pollutant distribution; therefore, heavy metals and PAH concentrations in sediments change in space and time (Everaert et al. 2017). It is critical to understand the persistence, bioaccumulation, toxicity, and chemical state of pollutants to carry out a proper remediation strategy.

### Most widespread and newest remedial technologies

Since a few decades ago, the environmental pollution caused by PAHs and heavy metals raised a growing interest among the world scientific community (Boehm 1964), mainly because of their high toxicity, low-degradability, bioaccumulation, and biomagnification along the food chains (Furness 2018; Shimada 2006; Khan et al. 2004; Nastro et al. 2014; Nastro et al. 2019). In order to solve the problem of marine pollution, different remediation techniques were developed over time. Generally, a remediation strategy must have as a primary objective the removal of pollutants or their transformation into less toxic compounds, with affordable costs. Degradation and/or transformation of pollutants into less dangerous compounds by means of chemical, physical, biological, or thermal methods are at the basis of remediation techniques. Among the in situ treatments, monitored natural recovery (MNR) is based on natural processes having the effect of containing, reducing, or eliminating the bioavailability or toxicity of contaminants (De Gisi et al. 2017). MNR is a non-invasive process and does not disrupt or destroy biologically active zones; it requires monitoring of the natural recovery process of an ecosystem over time (Fetters et al. 2020). Capping is a method alternative to MNR. It consists of a layer of clean material placed over the contaminated sediments to isolate the pollutants from the overlying water column and prevent the spread of pollutants throughout the water (Reible 2017). MNR and capping have the advantages of relatively low costs, reduced risks (usually associated with the transport and disposal of contaminated sediments), and a more limited impact on existing biological communities. However, contaminants are left in situ with the consequent risk of release and water pollution. Some chemical-physical treatments are applicable for sediments in situ remediation such as chemical oxidation, and immobilization/stabilization. These last ones consist of physical entrapment within a solid mass and/or use of chemically reactive materials for the segregation and/or degradation of contaminants by reducing their mobility, toxicity, and bioavailability (Majone et al. 2015). In addition to a low cost, they have the advantage of reducing the risk of resuspension and transport of contaminants, but the processes may be challenging to control. Ex situ techniques generally need a combination of several technologies to dredge or excavate, transport, treat, and dispose of sediment and residues. Sequential extraction techniques, especially applied for the remediation of sediments polluted with heavy metals, represent a good example of the ex situ remediation process (Mulligan et al. 2001; Okoro et al. 2012). According to their chemical-physical properties and pollution degree, sediments can undergo some of the following treatments: pre-treatment, physical separation processes, thermal extraction, bioleaching, electrolytic processes, solidification/stabilization, landfill confinement, vitrification, and chemical oxidation (Mulligan et al. 2001). Such treatments can reduce contaminants, provide short treatment times, require the use of equipment that can be easily installed, and allow the remediation of large areas. The main disadvantages concern the range of pollutant concentrations, the treatment efficiency, high management costs, and potential formation of toxic degradation by-products (Bhupendra and Pooja 2018)

In comparison with the physical and chemical processes, bioremediation is a more innovative and cost-effective technology, able to reduce pollutants concentrations in the marine environment with minimal impact (Nastro et al. 2014). In fact, microorganisms can use different metabolic pathways to degrade pollutants into less toxic forms (Qu et al. 2016; Babu et al. 2019). Remediation processes based on microbial metabolism has received much interest due to their reduced cost and environmentally friendly nature. In recent years, biological remediation processes proved their efficiency vs*.* several pollutants such as heavy metals and hydrocarbons, with encouraging results (Babu et al. 2019). Microbial-based techniques seem to be more promising for in situ treatment, such as biostimulation, bioventing, bioaugmentation (Babu et al. 2019). The recent extension of fuel cell utilization in bioremediation resulted in the set-up and development of a new bioelectrochemical technology, able to generate electricity from organic and inorganic substrates (heavy metal, PAHs, PCB, aldehydes/ketones, etc.) through bacterial metabolism (Logan 2006; Gambino et al. 2017; Santoro et al. 2017; Abbas et al. 2017a, b; Li and Yu 2015; Ghangrekar and Chatterjee 2017; Xia et al. 2015; Venkatesh and Pradeep 2016). Such systems are called sediment microbial fuel cells (SMFCs).

### Outline about bioelectrochemical systems and SMFCs

**Fig. 1:**
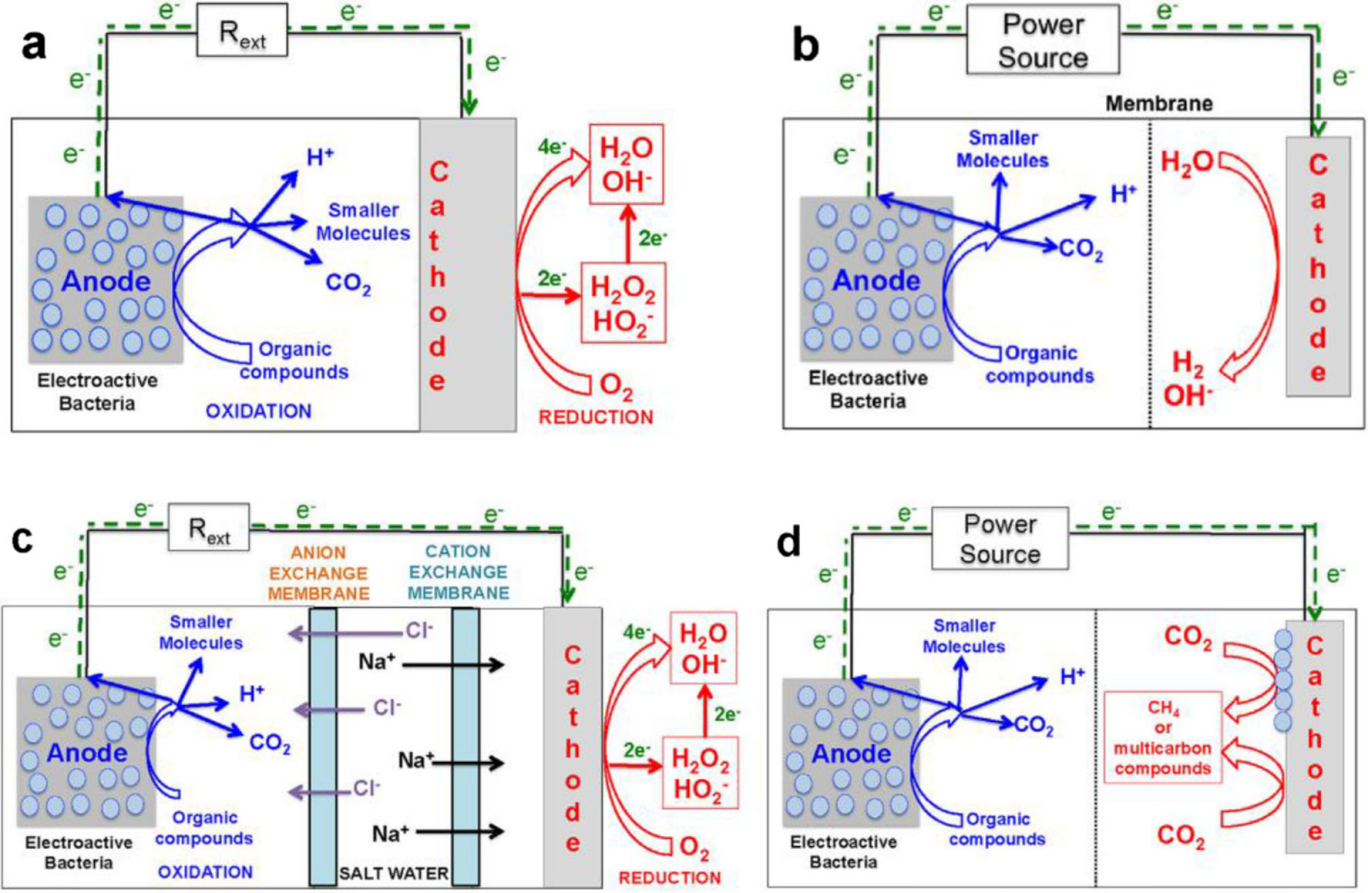
Overview on bioelectrochemical systems (BESs): single chamber, air cathode microbial fuel cell (MFC) (**a**), microbial electrolysis cell (MEC) (**b**), microbial desalination cell (MDC) (**c**), and general microbial electrosynthesis cell (MES) (**d**), reprinted from Santoro et al. 2017

Bioelectrochemical systems (BESs) represent an emerging technology whose application can range from industrial and municipal effluents treatment to water desalination, from the production of commodity chemicals and energy vectors to CO_2_ reuse (Santoro et al. 2017; Ghangrekar and Chatterjee 2017; Kadier et al. 2016; Avignone-Rossa and Nastro et al. 2019). BESs have the potential to reduce the environmental impacts of solid, liquid waste and agricultural systems management while contributing to energy saving by means of electric power and/or energy vectors production (Corbella et al. 2017; Li et al. 2018; Chandrasekhar et al. 2017; Florio et al. 2019; De Vrieze et al. 2018; Nastro et al. 2015; Flagiello et al. 2021). BESs include microbial electrolysis cells (MECs), microbial electrosynthesis cells (MES), microbial desalination cells (MDCs), all requiring an external source of energy, and microbial fuel cells (MFCs) which usually do not require any external energy input. MECs and MESs are mainly used to synthesise organic/inorganic molecules and MDCs to remove salts or other salty substrates from seawater. MFCs, instead, are mainly applied to wastewater treatment and sediment remediation even though further applications like the set-up of biosensors and innovative urban green infrastructures are being developed (Santoro et al. 2017; Endreny et al. 2020). In Fig. 1, we report a graphic representation of BESs according to Santoro et al. (2017).

All BESs are essentially based on the activity of exoelectrogenic bacteria, which, according to their physiology and the availability of mediators in the environment, can use both direct and mediated electron transport to exchange electrons with the electrodes (Huang et al. 2011a; Yasri et al. 2019). Such microorganisms are able to use both organic and inorganic molecules as a source of electrons and, for this reason, electrogenesis entails the degradation of organic compounds (pollutants included) or even the change of the redox state of metals. In this last case, metals can be accumulated in biofilm at the cathode or at the anode and, thus, removed from the environment (Abbas et al. 2017b; Fang and Achal 2019; Singh and Yakhmi 2014; Donovan et al. 2014; Wang et al. 2015). In comparison to other BESs, MFCs can work at environmental temperature and, generally, do not need an external source of energy if not for some peculiar applications like CO_2_ capture (Nastro and Avignone-Rossa 2019). In fact, they are based on biochemical processes naturally occurring at the electrodes in force of a difference in electrochemical potentials established between the electrodes (Wang et al. 2015; Nastro 2014; Logan et al. 2006).

**Fig. 2:**
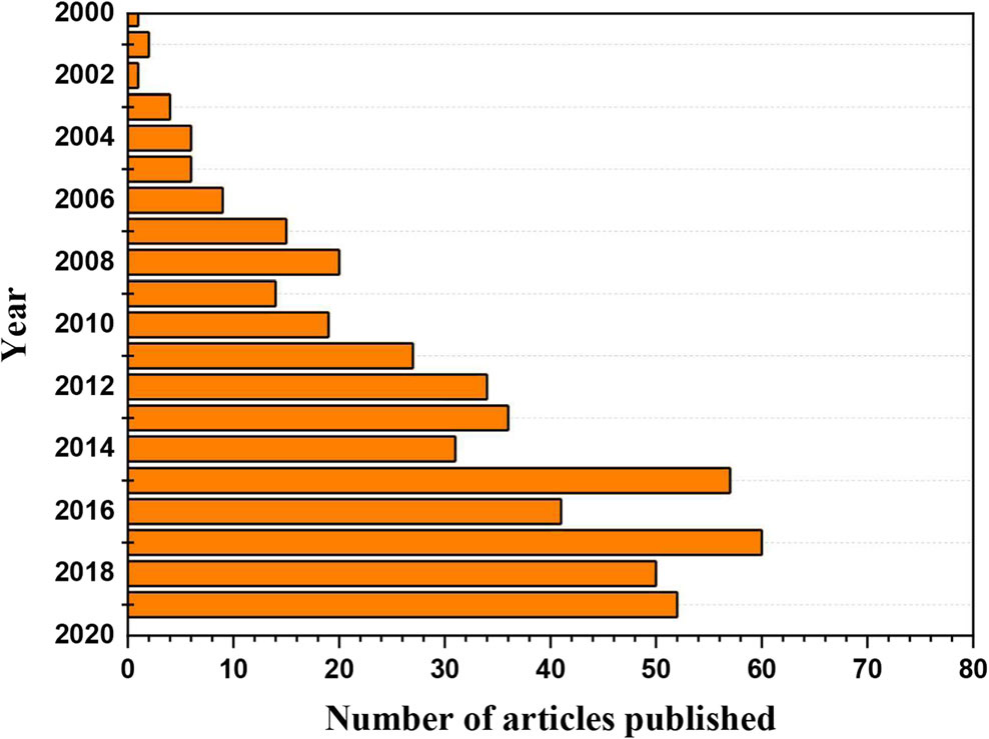
The number of articles published on sediment microbial fuel cells from 2000 to 2019 (the scientometric evaluation of the research on SMFC), including reaching 60 papers published in 2015

MFCs proved to be useful tools for the remediation of both water and sediments contaminated with heavy metals and/or hydrocarbons, with significant results even in view of an in-field application (Venkata Mohan and Chandrasekhar 2011b; Muhammad et al. 2016; Gambino et al. 2017; Wang et al. 2015; Nastro et al. 2019). When applied to environmental pollution treatment, the main outputs of MFCs consist in remediated water/sediment/soil and electric power. Laboratory-scale MFCs generally consists of an electrochemical cell with an anode, a cathode and an optional ion-selective membrane (as separator), and an external circuit for electron transport (Fig. 1a). Nevertheless, the application of MFCs to sediment remediation requires the set-up of a specific layout, making them different from other MFCs, so that researchers call them sediment-MFCs or SMFCs (Huang et al. 2011a; Gong et al. 2011; Abbas et al. 2017a; Rezaei et al. 2007). SMFC electric outputs are expressed as power density (PD) and current density (CD), usually referred to the anode or cathode surfaces as mW/m^2^ and mA/m^2^ (Wang et al. 2015; Sajana et al. 2016; Kronenberg et al. 2017). In few cases, PD is referred to m^3^ of remediated sediments (Morris and Jin 2012). Several studies have demonstrated that SMFCs succeed in both electricity generation and enhanced removal of persistent inorganic and organics from sediment (Alipanahi et al. 2019; Rezaei et al. 2007; Singh and Yakhmi 2014; We and You 2015; Nastro et al. 2019). Over the years, the interest of the scientific community towards the application of MFCs to sediment remediation has grown more and more. Since 2000, the number of papers published about SMFCs significantly increased, reaching 60 papers published in 2015 and 52 until 7th October 2019 (Fig. 2).

These data testify to the great interest obtained by SMFCs within the scientific community. In the year 2010, Yuan et al. (2010) made an attempt to construct a novel tubular air-cathode MFC to remove black colour and odour given by anaerobic bacteria to the organic-rich sediment in rivers. The provision of an electrode fostered the degradation of carbon-rich organic matter in the sediment, where the anode functioned as an electron acceptor. In this study, SMFCs achieved the highest power density of 107.1 ± 8.6 mow/m^2^, with 36% removal of readily oxidizable organic matter from sediments simultaneously. Chandrasekhar and Venkata Mohan (2012) designed and constructed an open-air cathode microbial fuel cell with non-catalysed graphite electrodes for the treatment of petroleum hydrocarbons. In this study, a maximum cell voltage of 343 mV (53.11 mW/m^2^) was achieved while treating complex petroleum hydrocarbons, with up to 2.49 g/l removal in 17 days of treatments. As to polycyclic aromatic hydrocarbons (PAHs), it was observed an increase of PAHs made up by 2-, 3-, and 4-rings in consequence of the degradation of aromatic compounds made up by with 5- and 6- rings. For example, dibenzo(A, H)anthracene and benzo(G, H, I)perylene showed nearly complete transformation (98 ± 1.2%) in comparison to the negative control (60 ± 6%), not provided with electrodes. Lee et al. (2015) created a separate water-layer by means of a fabric baffle for installing the anode electrode in sediment. SMFCs constructed with this methodology reached a maximum current density of 220.46 mA/m^2^, which is 3.9 times higher than control SMFCs. Nastro et al. (2019) applied SMFCs to marine sediments highly contaminated by PAHs. After 4 weeks of treatment, they obtained the removal of 17 μg/g of light PAHs (2–3 aromatic rings) and 9.79 μg/g of heavy PAHs (more than 4 aromatic rings). Li et al. (2020) constructed the SMFC in which cathode electrode was prepared with manganese dioxide/tourmaline composite (MnO_2_/T) material for efficient bioelectricity generation with concurrent waste remediation. SMFCs operated with MnO_2_/T cathode reported a higher power density of 368.99 mW/m^3^, which was 1.26 times higher than of SMFCs with MnO_2_ cathode. TOC and NH_4_^+^ removal were 55.7% and 93.6% respectively. Alipanahi and Rahimnejad (2018) evaluated the efficiency of the conductive and high surface containing metal brushes as a cathode electrode in the SMFC. In addition, they investigated the influence of diverse kinds of sediments (sea and three different areas of a river) in power generation of SMFC. Among all experimental conditions, the same researchers observed the highest power density of 121 μW/cm^2^ in SMFCs fed with river sediments thus proving that, such devices, can work in both marine and freshwater environments. All the studies as mentioned above suggest that SMFCs can be considered as devices for an efficient treatment of hydrocarbon and metal-contaminated sediments in both water habitats, while providing a source of renewable energy.

### SMFCs as a tool for in situ sediment remediation

**Fig. 3:**
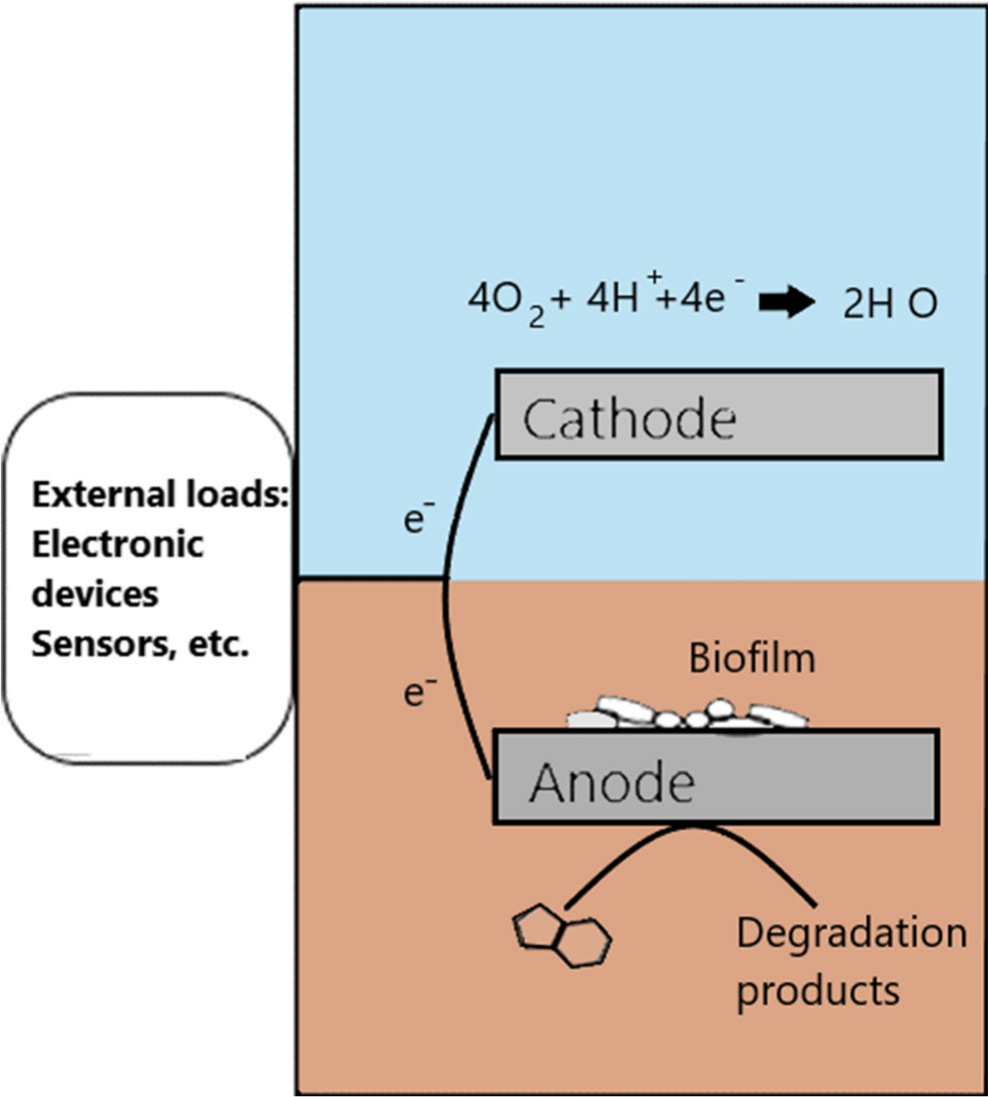
General scheme of an SMFC for PAHs degradation (Nastro et al. 2019). The anode, colonised by electroactive biofilm, is buried in sediments, and the electrons flow through an external electric circuit, reaching a cathode floating in the seawater. Organic compounds breakdown occurs at the anode

In the marine environment, bacteria are involved in several biogeochemical processes, entailing metal oxidation/reduction, mobilization from water to sediments and vice-versa and their assimilation by other organisms (Morel and Price 2003; Tagliabue et al. 2017; Nastro et al. 2014). Such metabolic activities allow microorganisms to detoxify polluted sediments while using metals as electron donors/acceptors in SMFCs (Lovley and Coates 1997). Several microbial species are able to use hydrocarbons (PAHs included) as a sole source of carbon (Nastro et al. 2014; Gambino et al. 2017; Barone et al. 2017; Cui et al. 2008; Wang et al. 2018). Nevertheless, natural assimilation of heavy metals and PAHs by microorganisms is slow processes because proper electron donors and acceptors, needed to foster such specific metabolic pathways, are very often lacking in sediments (Abbas et al. 2017b; Wang et al. 2018). In SMFCs, the electrodes can provide a less aggressive, inexhaustible, clean and flexible electron acceptor or donor in comparison to molecules available in the environment (Kronenberg et al. 2017; Xia et al. 2015). An SMFC typically consists of an anode made up of graphite or other carbon-based materials, and a cathode typically crafted with carbon-based materials or stainless steel. While the anode is buried in anaerobic/anoxic sediment, cathodes are placed in an oxygen-rich water phase (Erable et al. 2013; Mostafa Rahimnejad 2015; Kronenberg et al. 2017, Abbas et al. 2017b; Huang et al. 2011a, b; Nastro et al. 2019). As shown in Fig. 3, an electric circuit connects the electrodes. The same external circuit serves for energy harvesting. This energy can be used to power sensors or data logger to monitor the SMFC performance. A power management system is often connected to optimise SMFC operation as well (Nastro et al. 2019, Hongwei et al. 2015; Donovan et al. 2013).

SMFCs can be, at least in principle, easily placed in sea bottoms (Thomas et al. 2013) causing a minimal distraction for the aquatic habitats and they can work under a wide range of environmental conditions (Gong et al. 2011). Moreover, SMFCs can be set-up using low-cost materials (Huang et al. 2012) and require less frequent maintenance and simple construction techniques in comparison to other remediation methods (Tender et al. 2008). Despite the many advantages, SMFC technology has suffered some limitations like low operating voltages (Donovan et al. 2013) and unsuitableness to provide continuous power (Donovan et al. 2013; Yang et al. 2015). While designing and setting-up an SMFC, it is important to consider that its performance mainly depends on the gradient of electrochemical potential at the sediment-water interface (Malami et al. 2014), electrode materials and configuration (besides their respective distance), the abundance of electroactive bacteria at the anode, substrate availability, and sediment redox potential (Singh and Yakhmi 2014; Li and Yu 2015; Xia et al. 2015; Yang et al. 2015; Scott et al. 2008). As to microorganisms directly involved in electrogenesis, dissimilatory metal-reducing microorganisms seem to play an important role in SMFC operation (Yasri et al. 2019; Faisal et al. 2020). A great part of exoelectrogenic bacteria previously described belongs to α-, δ-, β-, γ- Proteobacteria as well as Firmicutes (*Clostridium* spp) and Archeobacteria (*Methanobacterium* spp and *Metanococcus* spp) taxonomic units (Wen-Wei and Han-Qing 2015; Abbas et al. 2017a, b; Faisal et al. 2020; Yasri et al. 2019). Due to the toxic environment, some aerobic microbes such as *Pseudomonas* spp., *Alteromonas* spp., *Acinetobacter* spp., and *Novosphigobium* spp., as well as some photosynthetic bacteria, colonise very often the cathodes (Wen-Wei and Han-Qing 2015; Abbas et al. 2017a, b; Faisal et al. 2020; Yasri et al. 2019; Erable et al. 2010; Milner et al. 2016).

**Fig. 4:**
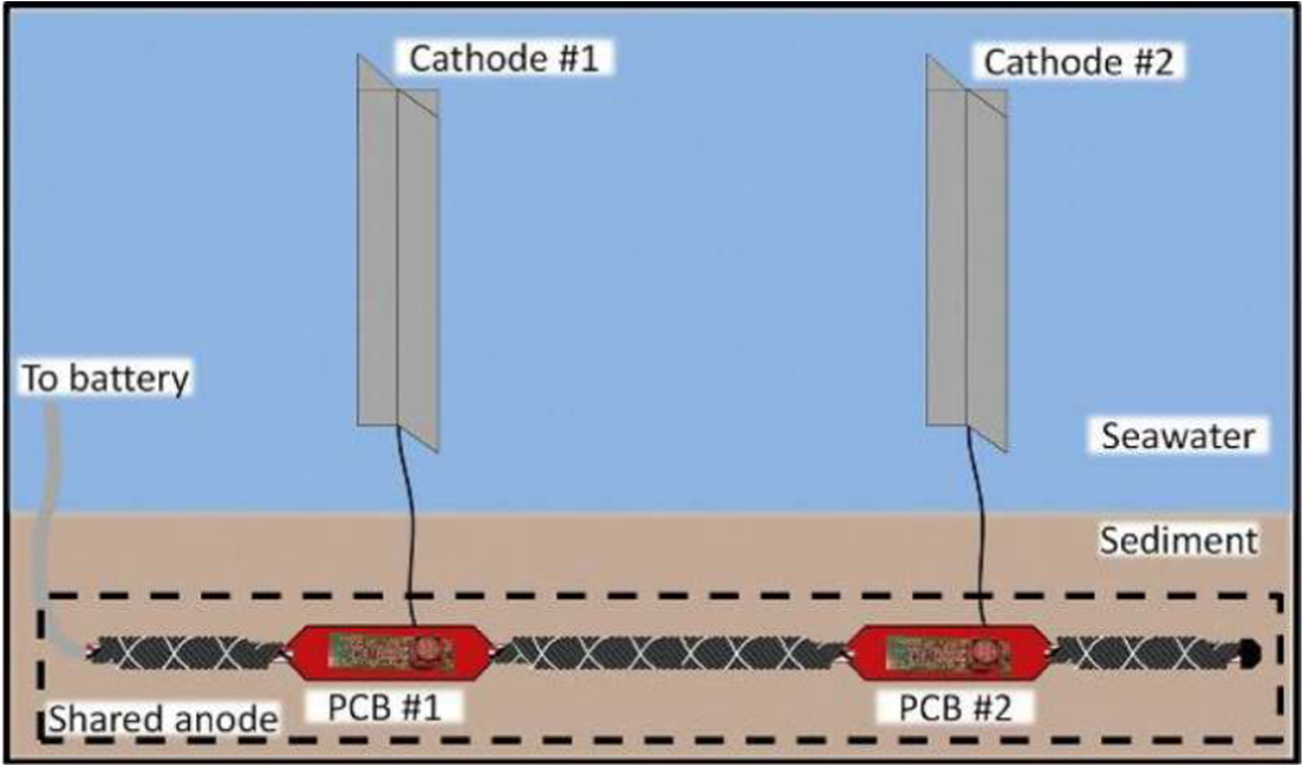
Example of scaled-up SMFCs by using multiple electrodes. A buried connection links several anodes while cathodes made-up of carbon fibres (1 m in length) (Babauta et al. 2018)

Intense research activities have been made to improve SMFC performance in lab-scale systems (Bao et al. 2017; Sayed Zaghum et al. 2017a; Henan et al. 2017; Kronenberg et al. 2017), with particular regard to the set-up and use of new materials in SMFCs. Further details about electrode materials are reported in section “SMFC materials in the marine environment: a challenge to win”). At the same time, new power management platforms, able to manage and/or store the energy produced, have been set up (Donovan et al. 2011; Liu et al. 2015; Alipanahi et al. 2019; Yang et al. 2015; Yamashita et al. 2019). The first SMFC prototypes date back to about 10 years ago (Gong et al. 2011; Guzman et al. 2010). Since then, SMFCs have been tested in sea rivers, lakes, and other aquatic environments (Yu and Li 2015; Li et al. 2017), with the aim to have a source of energy to feed sensors and other electronic devices for environmental monitoring (Hsu et al. 2013). However, great efforts have been spent to scale-up SMFCs to reclaim marine sediment polluted by PAHs (Song et al. 2014; Hong et al. 2010; Donovan et al. 2013b, Ewing et al. 2014; Zaisheng et al. 2012; Li et al. 2017, Babauta et al. 2018; Liu et al. 2016). Among the proposed solutions, maybe the most promising approach to the scaling up of SMFCs is through modularity, i.e. by setting up multi-electrode systems (Hsu et al. 2013; Xia et al. 2015; Babauta et al. 2018; Yang et al. 2015). According to Ewing et al. 2014, it could be possible to “electronically” scale-up SMFCs by using smaller-sized individually operated devices connected to a power management system that electrically isolates the anodes and cathodes, with significant improvement of power outputs: from 0.64 to 2.33 mW produced by single and four unite respectively. In any case, it is clear SMFCs can become competitive with other remedial technologies at as scaled-up systems. An example of scaled-up SMFCs, made up of a multianode system, is reported in Fig. 4 (Babauta et al. 2018). In it, each anode unit is connected to a cathode floating in seawater, where the oxygen acts as an electron acceptor. Overall, the system was composed of 20 m of a composite anode made by insulated twisted wire pair of copper conductors, as the core, wrapped with two layers of braided carbon yarn and titanium mesh acting as electron collector. Each anode unit was connected to two flyback converters, recharging a battery with a 77% efficiency (Babauta et al. 2018).

With the set-up of innovative power management systems, they proved their efficiency in pollutant removal and, at the same time, the ability to power electronic devices (sensors and wireless systems) and rechargeable batteries even for years (Liu et al. 2016; Babauta et al. 2018; Yang et al. 2015; Yamashita et al. 2019). As for the potential future commercialization, Trapero et al. (2017) carried out an economic assessment of MFCs for wastewater treatment. Their comparative analysis with activated sludge plants showed that MFCs could be a more attractive option with potential economic benefits even in the most pessimistic scenario. Let us consider that SMFC usually does not need pumping systems and has a less complex structure in comparison to MFCs for wastewater treatment. There is no reason to think that their commercialization could take a few advantages and profits in comparison to other remedial technologies. Nevertheless, the set-up of SMFCs on a large-scale will require a high level of engineering to place the anodes at a suitable depth under the sediments, the cathodes over the seabed surface, the electronic devices for SMFC management, and energy storage.

## MFCs as a tool for sediment remediation

### Hydrocarbons and metal removal in SMFCs: what mechanisms?

MFCs are self-induced potential-mediated bioelectrochemical devices, which can generate bioelectricity from a wide range of substrates, sediments, and dregs included (Abbas et al. 2017b; Fang and Achal 2019; Ghangrekar and Chatterjee 2017; Santoro et al. 2012; Zabihallahpoor et al. 2015). These last ones are formed by plant and animal detritus, dead microorganisms, faecal matter, and anthropogenic substances released in marine surroundings. All these are carbon-rich, biodegradable organic materials that can be consumed/treated by electrochemically active microorganisms to produce bioelectricity in SMFCs. In recent years, marine dreg remediation by SMFCs has been gaining much attention due to their low-cost and eco-friendly nature (Zabihallahpoor et al. 2015). Before discussing the hydrocarbon bioremediation mechanism, it is essential to determine how rapidly these pollutants can be biodegraded. Due to their highly hydrophobic nature, PAHs need to solubilise before being degraded by microorganisms. Hence, microorganisms and substrates play a crucial role in SMFC’s performance. In SMFCs, the metabolism of exoelectrogenic microorganisms induces an electrochemical potential acting as a driving force for the thermodynamically critical reactions. Such potential can be an alternate electron source for successful PAH remediation (Chandrasekhar et al. 2021; Zhang et al. 2010).The provision of solid electrodes, as in-exhaustible electron donors in the sediments, directly or indirectly boost the metabolic rate/substrate oxidation efficiency of electrochemically active microorganisms. With this phenomenon, complex PAHs in sediments can be transformed into less complicated compounds effortlessly (Chandrasekhar and Venkata Mohan 2012). For instance, Venkata Mohan and Chandrasekhar (2011) observed how heavy PAHs decreased at a higher rate in the area nearby an MFC anode than in the absence of electrodes in traditional anaerobic treatment. When the research about BESs started (about twenty years ago), a great part of the experimental activities was devoted to the production of hydrogen in MECs and bioelectricity from wastewater in MFCs (Logan et al. 2006; Santoro et al. 2017). In 2010, the first researches about MFCs applied to sediment remediation were published (Yuan et al. 2010; De Schamphelaire et al. 2010; Donovan et al. 2010; Erable et al. 2010; Guzman et al. 2010; Heijne et al. 2010; Hong et al. 2010; Zhang et al. 2010). With the help of 14C-labeling, Zhang et al. (2010) revealed that toluene could be biotransformed into carbon dioxide (CO_2_) in a *Geobacter metallireducens*-mediated MFC. Ever since the biotransformation/biodegradation of toluene was attained without the addition of an external electron acceptor other than the anode, it became clear how electrodes, in MFCs, can play as a solid electron acceptor and stimulate the biodegradation of PAHs in polluted anaerobic marine sediments. In a few words, anode serves as an alternate tool to overcoming electron acceptor limitations in PAH-contaminated marine sediments (Kronenberg et al. 2017). Electrochemically active microorganisms initially prefer to utilise light PAHs while they are involved in a catalytic breakdown of complex heavy PAHs into intermediate metabolites only under a favourable microenvironment. Initially, bacteria cause the saturation of at least one aromatic ring by fostering an anaerobic hydrolixation causing the formation of cis-dihydrodiol and the breakdown of PAHs (Chandrasekhar and Venkata Mohan 2012). These triggering reactions feed into the metabolic pathways that result in ring saturation and/or ring cleavage reactions, generating intermediate metabolites such as benzoyl-coA. Afterward, these intermediate metabolites are ultimately assimilated in biomass or fully oxidised (Varjani and Upasani 2017). In this anaerobic degradation process, instead of oxygen, microbes utilise alternative electron acceptors such as nitrate, iron(III), manganese(II), or CO_2_. In the case of SMFCs, anodophilic microorganisms are actively involved in the bioelectrochemical remediation of hydrocarbons via a series of metabolic reactions in the presence of electrodes acting as an inexhaustible acceptor. Electrochemically active anodophilic microorganisms initially oxidise hydrocarbons in the marine sediment by reducing the anode, whereas metal/sulphur-reducing bacteria oxidise anode generated S_elemental_ to SO_4_^2−^. Simultaneously an additional reaction that happens at the anode is the oxidation of S^2−^ to S_elemental_. While exoelectrogens oxidise petroleum hydrocarbons, oxygen, MnO_2_, Fe_2_O_3_, and SO_4_^2-^ are reduced among marine sediment surface layer and the anode (Nevin et al. 2011; Tender et al. 2008). The amount of reductants usually increase along with the sediment depth. The electrons produced by active biocatalysts in consequence of hydrocarbon oxidation can be, then, transported to the anode in two ways: by electrochemically active bacterial biofilm formed at the anode or by reduced ions (both in the form of dissolved and solid-phase), contained into sediments themselves (Zabihallahpoor et al. 2015). Several MFC configurations need electron mediators and proton exchange membrane (PEM) to transfer generated electrons to the surface of the anode and to transfer generated protons to the cathode chamber, respectively (Rahimnejad et al. 2012). Nevertheless, for SMFC operation, there is no need to provide PEM and electron mediators. Hence, we can consider SMFC-mediated bioelectrochemical treatment of polluted soil and sediment as an eco-friendly and low-cost substitute to traditional in situ bioremediation process.

The basic concept behind the metal removal in SMFCs lays into the reductive precipitation of metals acting as electron acceptors at the cathode in place of oxygen. The equations of the redox reactions occurring at the cathode are reported below (Eqs. 1–4):

1 $$ {\mathrm{Cr}}_2{\mathrm{O}}_7^{2-}\left(\mathrm{aq}\right)+6{\mathrm{e}}^{-}+14{\mathrm{H}}^{+}\kern0.5em \to \kern0.5em 2{\mathrm{Cr}}^{3+}\left(\mathrm{aq}\right)+7{\mathrm{H}}_2\mathrm{O} $$

2 $$ {\mathrm{Co}}^{2+}\left(\mathrm{aq}\right)+2{\mathrm{e}}^{-}\kern0.5em \to \kern0.5em \mathrm{Co}\left(\mathrm{s}\right) $$

3 $$ {\mathrm{Cu}}^{2+}\left(\mathrm{aq}\right)+2{\mathrm{e}}^{-}\kern0.5em \to \kern0.5em \mathrm{Cu}\left(\mathrm{s}\right) $$

4 $$ {\mathrm{Hg}}^{2+}\left(\mathrm{aq}\right)+2{\mathrm{e}}^{-}\kern0.5em \to \kern0.5em \mathrm{Hg}\left(\mathrm{s}\right) $$

**Fig. 5:**
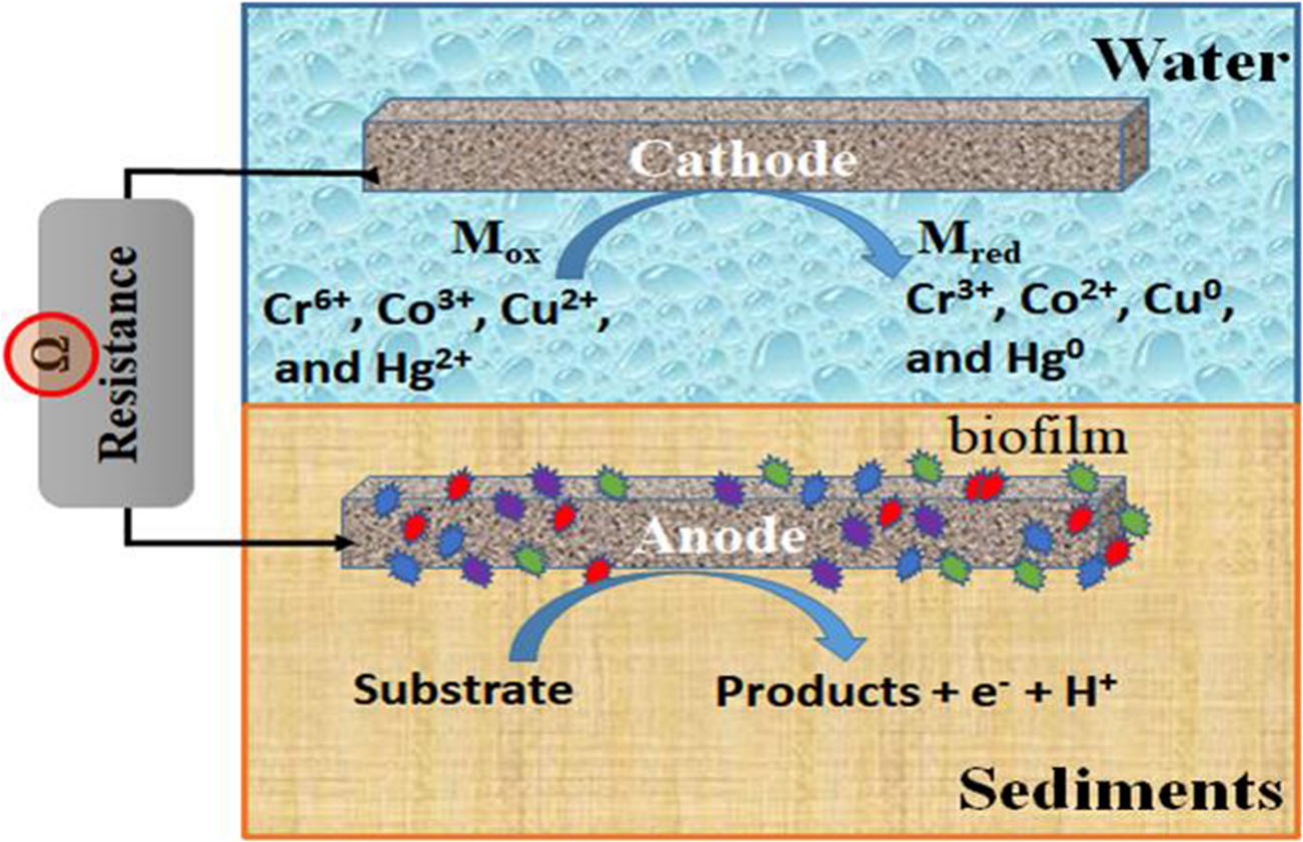
General scheme of an SMFC for metal removal. Heavy metal cations act as electron acceptors at the cathode, changing their oxidative state

The electrons needed for metal reduction are released during the biotransformation of the substrate (mainly organic compounds) by electrochemically active bacteria at the anode, thus providing the driving force for a bioelectrochemical metal reduction at the cathode (Nancharaiah et al. 2015; Wang et al. 2011). Therefore, redox reactions involving Cr(VI), Co(III), Cu(II), and Hg(II) occur at the cathode while the bacteria at the anode carry out the breakdown of organic compounds. A general scheme of SMFCs for heavy metal removal from sediments and cathodic reactions during metal removal/recovery in SMFC is reported in Fig. 5.

According to their chemical properties, the reduced metals either form deposits on the cathode surface, precipitate in the electrolyte solution (sediment), or stay soluble in the sediment. The reduction of metal ions at the cathode comes to be spontaneous due to the difference in electrochemical potentials established at the cathode and the anode. This difference has a positive value if metal ion reduction potentials are higher than the anode (Nancharaiah et al. 2015). Hence, the metal ions like Cr(VI), Co(III), Cu(II), and Hg(II), which are having a positive redox potential, can be removed from the sediments by the reductive precipitation process in SMFCs (Heijne et al. 2010; Huang et al. 2014; Wang et al. 2008; Wang et al. 2011). Nancharaiah et al. (2015) and Fang and Achal (2019) report further information about the mechanisms at the basis of metal removal by SMFCs. As we discussed earlier, this entire metal removal process in SMFC is associated with substrate oxidation/organic removal from sediments, with simultaneous electricity generation (Nancharaiah et al. 2015). As a result, SMFC offers a unique platform for bioelectrochemical remediation of pollutants and toxic metals through redox reactions.

## Systems efficiency: energy balance

When we talk about MFC efficiency, we refer to “coulombic efficiency” (CE), i.e. the percentage of energy stored into chemical compounds and converted into electric power by exoelectrogenic bacteria (Logan et al. 2006). For in-batch systems fed with wastewater or with solutions containing a well-known amount of substrate (acetate, glucose, glycerol, etc.), CE calculation can be easily performed by measuring the variation in COD content (or in a given compound concentration) and the current produced along with MFC operation. In SMFCs, especially in the case of an in-field remediation, it is not that easy to evaluate CE. Besides the specific pollutants to be removed, bacteria can use other substrates already present in the sediments to sustain their metabolism and, then, the electrogenesis. Therefore, it would be very difficult to calculate the percentage of pollutants (a given PAH or PCB for example) directly converted into electricity. Moreover, in aquatic environments, there is a constant fall of organic matter from the water column. COD removal rate by SMFCs should take into account the speed of precipitation of the OM particles towards the sediment and their removal in the area nearby the anodic electrode, as reported in González-Gamboa et al. (2018). So, most part of the authors, while discussing SMFC performance, refer to cell voltage, CD and PD produced and percentage of pollutants removed rather than CE. In a few cases, an approximate calculation of CE is reported and, to the best of the authors’ knowledge, it rarely overcomes 20% (Yang et al. 2010). It is important to underline that SMFCs are aimed at remediating rather than producing electric power. Even though the energy outputs are low, it has been estimated that, among all MFCs, SMFCs could be the only bioelectrical system able to pay back the energy spent for their set-up, with a minimum of 2.7 years and a maximum of 8.5-year duration (Tommasi and Lombardelli 2017; Yang et al. 2015). According to other authors, such systems might last even multiple decades while powering wireless sensor network (Guzman et al. 2010). In their work, Tommasi and Lombardelli (2017) suggested the following formula (Eq. 5) to calculate the energy produced by a SMFC:

5 $$ {E}_{MFC}=\underset{t\mathrm{o}}{\overset{t}{\int }}I\cdotp \Delta V\cdot dt $$

where *I* is the current intensity produced over a well-defined period of time, Δ*V* is the difference in electrochemical potentials established between the electrodes, and *E* is the energy expressed in Joule. According to Tommasi and Lombardelli (2017), the net energy produced by MFCs could be obtained by calculating the difference between the energy produced under the form of generated electricity when the external circuit is closed (*E*_MFC_) and the energy spent to set-up and maintained the system, for example, to oxygenate the cathode (namely the direct energy, *E*_Direct_ ) (6).

6 $$ {E}_{net}={E}_{MFC}-{E}_{direct} $$

*E*_MFC_ is, in a few words, the electrical energy produced from substrates oxidation and it is affected by several factors: substrate nature, MFC operating conditions, and the abundance of the electrochemically active microbial population at the anode, so *E*_MFC_ amount can be indeed variable. As a general statement, the energy balance can be positive or negative whether an MFC is able or not to repay the energy used to set-up and operate the device itself. Therefore, SMFCs can produce a positive *E*_net_ when the energy generated is higher than the amount used to operate the system over time (Tommasi and Lombardelli 2017; Yang et al. 2015). If that is the case, then the *E*_net_ can be used to provide intermittent aeration at the cathode or to light up LED bulbs, operate sensors to measure the chemical-physical state of marine water stream and other low-power electronic devices (Guzman et al. 2010; Gong et al. 2011; Liu et al. 2015). Recently, the possibility of utilization of photosynthetic microorganisms at the cathode has been proposed to improve the performance over time, avoiding external energy inputs (Erable et al. 2013; Luimstra et al. 2014). The net energy calculation can represent a reference parameter to compare SMFCs to other remedial technologies. Usually, the energy costs of remediation treatments at the point of use are expressed as kWh/m^3^ of treated sediments. At the present stage of development of SMFCs, the energy produced is expressed as Wh per m^2^ of cathode/anode surface, so a direct comparison is not applicable because of the different units of measure adopted. As a practical example, the results obtained by Bianco et al. (2020), who implemented an in-lab anaerobic bioremediation process of marine sediments polluted with Phenanthrene, coupled with methanogenesis, showed an energy demand of 16 kWh per m^3^ of treated sediments for a treatment lasted 42 days, with the removal of 68% in the best case. By setting up lab-scale SMFCs with aerated cathodes to remediate marine sediments heavily contaminated by PAHs, the authors of this paper estimated an approximate energy demand of 1.05 kWh per m^2^ of anode surface for an operation period of 28 days. At the end of the experiments, PAH removal in SMFCs ranged from 86% of naphthalene to 10% of Indenol[1,2,3-c,d]pyrene, with a 67% removal of phenanthrene (Nastro et al. 2019). If the efficiency of both methods can be compared in terms of sediment remediation, the anaerobic bioremediation and SMFCs cannot be directly compared in terms of process energy demand. Life cycle assessment (LCA) by using MJ of primary energy and evaluating the secondary energy needed for certain processes can be a useful tool to assess the energy costs of remediation processes and carry out a comparison among them, even in terms of environmental impacts (Visentin et al. 2019; Ulgiati et al. 2011; Puccini et al. 2013). As SMFCs is a new technology at the edge of an in-field application, no studies are already available, while LCA has been applied to other BESs, with some encouraging results (Corbella et al. 2017; Garbi et al. 2017; Zhang et al. 2019; Pandit et al. 2020). We believe such an approach will be the best one to compare SMFC technology with other remedial techniques and help decision-makers in their choices.

## SMFC materials in the marine environment: a challenge to win

Any device working in a marine environment has to deal with the corrosive action of dissolved salts and electrodes are no exception. Electrodes fouling, with a consequent decrease in power outputs, can heavily affect SMFC performance. For this reason, the choice of electrode materials is of fundamental importance (Fang and Achal 2019; Mustakeem 2015; Tommasi et al. 2016) because they have to withstand corrosion in a long-term period (Yaqoob et al. 2020). Biocompatibility is a prerequisite for electrode materials, it is not possible to use any antifouling coating treatments so, the transition from lab-scale to an in-field utilization of SMFCs cannot exempt from an accurate preliminary study and testing of materials to be used in scaled-up devices. Revamping electrode configurations and the use of novel materials have also to be taken into account while setting-up scaled SMFCs (Zhang et al. 2011). If the first and one of the favourite material electrodes is graphite, advances in research led to the use of other carbon-based materials (Babauta et al. 2018; Scott et al. 2008), which can be eventually combined to improve the overall power outputs (Liu et al. 2015). For their properties, carbon-based materials are the most frequently used to create anodes. Granular activated carbon (GAC), carbon nanotubes (CNTs), graphite fibre brushes, carbon cloth, carbon paper, carbon yarn, reticulated, and carbon felt are some examples (Wei et al. 2011; Jiang and Li 2009; Karra et al. 2014; Tommasi et al. 2016; Sudirjo et al. 2019; Santoro et al. 2017; Patel et al. 2019; Thepsuparungsikul et al. 2014; Babauta et al. 2018). These materials are chosen for their stability in microbial cultures, high electric conductivity and specific surface area, micro-porosity, biocompatibility, chemical stability, high resistance to corrosion, and they are, generally, of affordable cost (Wei et al. 2011; Jiang and Li 2009; Yakoob et al. 2020; Karra et al. 2014; Fang and Achal 2019; Tommasi et al. 2016; Sonawane et al. 2017). Recently, the use of graphene-based materials, natural and recycled materials, and synthetic materials has been tested in MFCs for various applications (Sonawane et al. 2017) but at the best of our knowledge, not in the marine environment. In Table 1, we report the main features of carbon-based materials used at the anode. Baudler et al. (2015) tested metal anodes in MFCs, with copper suitable for application in high-outputs BESs. Nevertheless, any utilization of metals in the marine environment has to take into account the corrosion issues. Among metals, stainless steel (SS) and carbon steel (CS) can be good candidates for electrodes set-up in the marine environment (Erable et al. 2010). CS is widely used for applications in the marine environment: it has good mechanical characteristics, but it can go through fouling episodes (Reimers et al. 2006; Refait et al. 2020). Among all carbon-based materials, nanotubes (CNTs) and carbon steel (CS) seem to be quite promising for constructing anodes for an infield application of SMFCs.

**Table 1:** Different types of materials used for SMFC electrodes (most of the materials used as an anode can be used as a cathode)

Electrode materials	Advantage	Disadvantage	References
Activated carbon (AC)	•Durable •High surface area •Low cost •High micro-porosity •Catalytic activities •High conductivity	•Critical factor for scaling up •Biocompatibility issue	Karra et al. 2014. Muhoza et al. 2017 Chatterjee et al. 2018
Carbon felt (CF)	•High electrical conductivity •High porosity •Low cost •High mechanical strength •Wide surface area	Large resistance	Santoro et al. 2017 Deng et al. 2009 Chatterjee et al. 2018
Carbon brush (CB)	•High surface area •Easily emplaced in sediments •Biofilm growth •Easy construction	Clogging	Sonawane et al. 2017 Chatterjee et al. 2018
Carbon nanotubes (CNTs)	•Good cell adhesion •Good catalytic activities •High specific area •Corrosion resistance	Large resistance	Heister E. et al. 2013 Chatterjee et al. 2018
Carbon steel (CS)	•High conductivity •Relatively cheap •Easy accessibility •Wide used for marine applications	Low surface area	Refait et al. 2020 C. Dumas et al. 2007 Chatterjee et al. 2018
Graphite	•Good electrical conductivity and chemical stability •Relatively inexpensive •Easy accessibility	Surface area difficult to increase	Zhou et al. 2011 Chatterjee et al. 2018 Sonawane et al. 2017

Cathodes in SMFCs float in overlying marine waters, so materials used to create these electrodes should have high mechanical strength, besides proper catalytic performance and resistance to corrosion. A wide range of materials has been tested to improve the power outputs of SMFCs: carbon felt, carbon brush, carbon fibre and titanium wires, GAC, graphite, stainless steel (Deng et al. 2009; Dumas et al. 2007; Karry et al. 2015; Babauta et al. 2018). Dumas et al. (2007) carried out a comparison between graphite and stainless steel to design efficient cathodes in SMFCs. In their work, they found out that graphite cathode correlated with higher open-circuit potential in comparison to stainless steel electrodes and resulted too brittle for scaling up. On the contrary, compact cathodes with a large surface area and a proper structure can foster oxygen reaction with protons and electrons. Bergel et al. (2005) compared carbon fibre and coated stainless steel cathodes in an SMFC, revealing higher effectiveness of carbon fibres in terms of overall SMFC performance; nevertheless, stainless steel showed better mechanical features. Lately, Mustakeem (2015) suggested the utilization of doped carbon materials to improve SMFC power outputs. Platinum is the most successful catalyst for oxygen reduction, but its high cost, pH sensitivity, sulphide poisoning, and non-sustainability, as well as its short-term activity in the presence of salts or impurities, limit its use in commercial applications (Bergel et al. 2005; Zhang et al. 2009; Cheng et al. 2006; Reimers et al. 2006). Therefore, non-Pt-based catalysts were developed as an alternative, for example, cobalt tetramethyl phenyl porphyrin (CoTTP), manganese oxides, and cerium can be used, with interesting results in terms of performance and potential costs (Yasri et al. 2019; Reimers et al. 2006; Imran et al. 2019). Other materials that have been tested at the cathode of MFCs are composite compounds, i.e. metal macrocyclic compounds such as iron phthalocyanines or CoTTP, nitrogen-doped carbon materials, and electroconductive polymers, graphene-modified polyacrylonitrile fibre, all with good performance (Yasri et al. 2019; Wang et al. 2019). For in-field application, biocatalysis represents an alternative to the use of platinum or other metals (De Schamphelaire et al. 2010; Santoro et al. 2010; He and Angenent 2006). Biocathodes are more advantageous than abiotic cathodes, as they are economically sustainable, have comparable performance as cathodes coated with expensive catalysts (e.g. Pt), and can provide a promising self-sustained, free to use, stable alternative to chemical catalysts for oxygen reduction reactions in MFCs (Milner et al. 2016). For example, biofilm-coated stainless-steel cathodes are very promising candidates for implementation in marine MFCs, as the cathode progressively acquires effective catalytic properties and it is less vulnerable to corrosion (Huang et al. 2011a, b).

## Future scope of SMFC

It is an essential step to take necessary actions to reduce the low power densities in SMFCs by altering the existing reactor design and reduce the losses affected by activation, ohmic, and concentration overpotentials (Nastro et al. 2015). It is essential to prevent the losses caused by unnecessary reactions, such as direct oxidation of the substrate (PAHs or metals) in the presence of O_2_ near the anode electrode, which will reduce the overall efficiency (power generation and pollutant remediation) of the SMFC. Furthermore, recent studies suggesting that maintaining an optimum distance between the electrodes (anode and cathode) is required to reduce the concentration overpotential. The anode electrode must properly be submerged in the sediment to avoid O_2_ diffusion towards the anode surface (Venkata Mohan and Chandrasekhar 2011; Chandrasekhar et al. 2017). Furthermore, increasing the electrode size in SMFCs will lead to a drop in power density, which suggests that SMFCs do not scale up with size (Hsu et al. 2013; Ewing et al. 2014). As an alternative to increasing in size, an electronic scaling up was suggested by Ewing et al. (2014) through the setting-up of mini SMFCs connected to a power management system, able to electrically insulate the anodes and the cathodes. This approach led to an increase in SMFC performance on a long-term basis in comparison to single units. The same authors suggest this could be the best way to enhance the overall process efficiency (Ewing et al. 2014). Other stacked approaches remain to be explored. It is also necessary to take proper precautions to grow an electrochemically active bacterial population on/around the anode surface. It can possibly be achieved by bioaugmentation of anodophilic bacteria and possible field effects due to anode morphology and conductivity (Chandrasekhar and Venkata Mohan 2012; Chandrasekhar et al. 2017). Constant efforts are being made to create better electron transfer among the anode and the anodophilic microorganism by altering anode surfaces and coating an active catalyst on the surface of the electrode (Chandrasekhar 2019). SMFC technology is facing many challenges to become a renewable energy source and an efficient bioremediation technique. Therefore, future researches should address the bottlenecks affecting SMFC performance and potential impacts on ecological systems. The future development of the SMFC requires the efforts of scientists from many fields such as ecology, microbiology, computer science, electrochemistry, engineering, and materials science, but the route is already open.

## Conclusions

The constant introduction of pollutants in the marine environment, despite the many regulations at both national and international levels, puts the ecosystems as well as human health at risk. Many remedial techniques are already available and, recently, SMFCs have been gaining the interest of scientist all over the world for their ability to combine environmental remediation and renewable energy production. Nevertheless, this newborn technology has just moved the first steps towards an in-field application, with the first pilot plants tested. Even so, there are still some bottlenecks to overcome. The choice of materials to set-up the SMFCs themselves and the approach to the scaling up and the set-up of energy harvesting systems remain among the most relevant issues to address. In future, the bioelectricity generation, with subsequent sediment remediation, will help in pay reimbursement for the cost of the remediation process, making it affordable and, given the double nature of SMFCs (remedial devices and renewable energy generators); a LCA approach will be an appropriate tool to compare SMFCs with other remedial techniques

## Data Availability

Not applicable
